# Supplementary material for: Infant Milk Formula Enriched in Dairy Cream Brings Its Digestibility Closer to Human Milk and Supports Intestinal Health in Pre-Clinical Studies
Source: Nutrients. 2024 Sep 11;16(18):3065. doi: 10.3390/nu16183065 (PMC11434767; doi:10.3390/nu16183065)
Supplement: Supplementary file 1 [file nutrients-16-03065-s001.zip › nutrients-3186431-supplementary.pdf]

Supplementary Materials

Infant Milk Formula Enriched in Dairy Cream Brings Its Digestibility Closer to Human Milk and Supports Intestinal Health in Pre-Clinical Studies

Alina Kondrashina 1,*, Gianfranco Mamone 2, Linda Giblin 3 and Jonathan A. Lane 1,*
* Correspondence: alina.kondrashina@hh.global (A.K.); jonathan@hh.global (J.A.L.)

S1. MATERIALS AND METHODS

S1.1 Proteomics

Samples obtained after gastric and intestinal digestion were lyophilized and dissolved in 1 mL of milli-Q water, alongside apical and basolateral supernatants after absorption. An aliquot (50 µL) was desalted by spin-column C18 (Millipore) and the eluate was dried by speed-vac and then reconstituted in 100 µL formic acid 0.1%. Desalted samples (5 µL) were analysed by liquid chromatography tandem mass spectrometry (LC-MS/MS).

The nano LC-MS/MS analyses were performed using an Ultimate 3000 nanoflow ultrahigh performance liquid chromatography (Dionex/Thermo Scientific, San Jose, CA) coupled to a Q Exactive Orbitrap mass spectrometer (Thermo Scientific). Samples were loaded through Acclaim PepMap 100 trap columns (75-µm i.d. × 2 cm; Thermo Scientific) using a FAMOS autosampler (Thermo Scientific). Eluent A was 0.1% formic acid (v/v) in LC−MS grade water; eluent B was 0.1% formic acid (v/v) in 80% aqueous acetonitrile. Peptides were separated using an EASY-SprayTM PepMap C18 column (25 cm × 75 µm) with 2 µm particles and 100-Å pore size (Thermo Scientific), and a 2–50% gradient of B over 40 min after 10 min of isocratic elution at 2% B, at a constant flow rate of 300 nL/min. MS1 precursor spectra were acquired in the positive ionization mode scanning the 1,600–250 m/z range with resolving power of 70,000 full width at half maximum (FWHM), an automatic gain control (AGC) target of 1 × 106 ions, and maximum ion injection time of 120 ms. The spectrometer operated in full scan MS1 and data-dependent acquisition mode, selecting up to the 10 most intense ions for MS/MS fragmentation and applying a 10-s dynamic exclusion. Fragmentation spectra were obtained at a resolving power of 17,500 FWHM. Ions with charge greater than +6 were excluded from the MS/MS fragmentation. Spectra were elaborated using the software Xcalibur version 3.1 (Thermo Scientific). Each sample was analysed in triplicate (technical replicate).

Raw files of the nano LC−MS/MS runs were used as the output for protein identification using the Andromeda search engine of the open source MaxQuant bioinformatic suite (version 2.0.3.0) against the Bos taurus milk protein database (αs1-casein, αs2-casein, β-casein, κ-casein, β-lactoglobulin, α-Lactalbumin) (Uniprot). Search parameters were as follows: mass tolerance value 8 ppm for the precursor and 0.02 for the fragment ions, unspecific cleavage; Met oxidation, pyroglutamic acid at N-terminus Gln, Ser/Thr phosphorylation at serine and threonine, and Hex and Lac glycosylation at lysine residues, as variable modifications. Peptide spectrum matches were filtered using the target decoy database approach with an e value of 0.01 peptide-level false discovery rate (FDR), corresponding to a 99% confidence score

SUPPLEMENTARY TABLES

Table S1. Number of unique peptides identified in digested samples of LC-IMF and HC-IMF and sequence coverage of their original proteins.

	**Unique peptides**		**Unique sequence coverage [%]**	
	LC-IMF	HC-IMF	LC-IMF	HC-IMF
**Alpha-lactalbumin**	31	62	45.8	48.6
**Alpha-S1-casein**	192	424	93.5	94.9
**Alpha-S2-casein**	92	311	61.3	90.5
**Beta-casein**	280	647	92.4	93.3
**Kappa-casein**	110	251	75.8	88.4
**Beta-lactoglobulin**	117	381	68.5	82

Table S2. Free AAs released in the course of simulated infant gastrointestinal digestion.

		Cysteic acid	Taurine	Asp	Thr	Ser	Glu	Gly	Ala	Cys	Val	Met	Ile	Leu	Tyr	Phe	His	Lys	NH3	Arg	Pro
G0	**LC-IMF**	125.64	161.12	<LOD	<LOD	<LOD	59.49	18.69	12.61	403.16	<LOD	<LOD	14.23	<LOD	538.84	<LOD	16.45	10.83	439.26	7.02	23.74
	**HC-IMF**	110.61	<LOD	10.95	<LOD	3.03	71.26	21.26	<LOD	444.37	<LOD	7.99	3.41	2.72	508.31	<LOD	6.58	8.80	417.69	<LOD	28.03
	**HBM**	41.54	143.79	42.53	269.88	104.21	1734.68	113.47	198.02	34.51	42.56	1.84	6.41	12.74	<LOD	14.03	24.05	9.64	549.20	8.20	<LOD
G60	**LC-IMF**	40.46	109.21	<LOD	<LOD	<LOD	27.47	10.00	<LOD	231.36	<LOD	<LOD	<LOD	<LOD	296.87	<LOD	69.38	<LOD	320.95	<LOD	<LOD
	**HC-IMF**	79.40	273.12	<LOD	13.21	<LOD	39.25	18.50	4.01	276.80	6.85	<LOD	<LOD	<LOD	243.24	<LOD	97.83	6.48	579.75	<LOD	12.77
	**HBM**	42.48	130.92	23.85	20.18	53.83	1130.31	70.96	135.20	23.67	36.59	7.10	15.03	123.90	2.77	17.55	25.81	14.87	487.79	10.58	<LOD
I60	**LC-IMF**	833.57	715.13	141.76	299.18	305.99	420.66	379.99	397.05	209.51	501.78	250.76	268.44	1485.07	1309.68	1905.91	1069.69	2123.81	874.57	1125.05	37.80
	**HC-IMF**	856.57	694.19	148.60	304.67	308.66	577.96	379.92	280.12	251.72	506.32	295.32	218.27	1343.49	1211.28	1011.52	945.24	2049.40	1706.56	1115.96	33.86
	**HBM**	174.90	165.11	192.61	246.94	480.03	1562.45	433.57	702.32	90.73	511.37	289.44	417.16	1479.98	633.80	689.70	348.98	1370.09	848.01	690.94	53.03
Apical	**LC-IMF**	71.97	13.05	39.20	321.71	249.39	234.34	125.82	345.02	53.13	320.54	101.68	251.42	604.51	234.58	219.56	118.09	473.55	204.79	210.21	216.65
	**HC-IMF**	88.65	29.65	37.48	310.41	240.87	228.31	123.83	333.14	51.47	310.73	99.36	244.02	585.43	222.99	209.38	113.68	452.77	204.92	197.68	223.08
	**HBM**	14.88	14.71	49.90	251.95	193.65	332.82	137.14	261.34	36.51	231.61	59.26	166.78	398.62	118.41	136.11	75.31	293.89	215.47	130.93	218.09
Baso	**LC-IMF**	2.49	15.17	1.27	3.48	3.34	5.81	3.09	8.25	4.04	4.91	1.25	4.87	4.29	1.05	3.67	10.28	6.10	8.81	6.60	<LOD
	**HC-IMF**	3.22	14.67	0.78	4.44	3.37	5.17	3.92	9.67	4.63	6.62	2.50	5.43	5.86	0.74	3.37	9.32	8.04	12.33	8.04	<LOD
	**HBM**	2.46	13.81	0.61	3.29	3.12	4.26	2.99	8.51	3.93	3.51	0.40	3.15	3.70	<LOD	2.57	7.28	4.07	25.48	7.27	<LOD
